# Factors affecting anxiety and depression during the first wave of the COVID-19 pandemic: a cross-sectional study of three different populations

**DOI:** 10.1186/s41983-022-00510-9

**Published:** 2022-06-11

**Authors:** Cemile Hurrem Ayhan-Balik, Seda Karakaya, Fatma Yasemin Kutlu

**Affiliations:** 1 Department of Psychiatric Nursing, Faculty of Health Science, Van Yuzuncuyıl University, Van, Turkey; 2 Sultan 2.Abdul Hamid Khan Educational and Research Hospital, Istanbul, Turkey; 3 Department of Mental Health and Psychiatric Nursing, Florence Nightingale Faculty of Nursing, Istanbul University-Cerrahpasa, Istanbul, Turkey

**Keywords:** COVID-19, Pandemic, Mental health, Anxiety, Depressive symptoms, Cross-sectional study

## Abstract

**Background:**

This paper was the first study comparing levels of anxiety and depression and assessing the affecting factors among the general population, frontline healthcare workers, and COVID-19 inpatients in Turkey during the first wave of the COVID-19 pandemic. We collected data from the general population (*n* = 162), frontline healthcare workers (*n* = 131), and COVID-19 inpatients (*n* = 86) using Individual Characteristics Form, Generalised Anxiety Disorder Scale, and Beck Depression Inventory in this cross-sectional study.

**Results:**

An increased prevalence of depression and anxiety were found predominantly in frontline healthcare workers (*p* < 0.001). COVID-19 inpatients and frontline healthcare workers were more likely to demonstrate anxiety (*p* < 0.001) than the general population. In the regression analysis, while fear of infecting relatives was a significant predictor of anxiety and depression in the general population, gender and experiencing important life events were associated with anxiety. Fear of infecting relatives and lack of personal protective equipment while providing care were predictors of anxiety and depression in healthcare workers (*p* < 0.001). Furthermore, the fear of being re-hospitalised due to re-infection was a predictor of depression and anxiety levels of the COVID-19 inpatients.

**Conclusion:**

Policymakers and mental health providers are advised to continuously monitor psychological outcomes and provide necessary health support during this pandemic.

## Introduction

The novel coronavirus (2019-nCoV) was first detected in Wuhan, China, at the end of 2019. It spread rapidly to other countries all over the world. On March 11, 2020, the World Health Organization (WHO) declared the COVID-19 outbreak a pandemic and public health emergency of international concern [35]. Since March 2020, strict preventive measures have been taken by governments worldwide. At the time of writing this article, there were over 200 million confirmed COVID-19 cases and 4.32 million deaths as a result of the disease globally. The number of confirmed cases in Turkey was reported at 12,051,852 in February 2022. Turkey’s fourth wave of COVID-19 infections was reported in February 2022 [21].

The number of cases has decreased with the development of the vaccine. Still, problems persist in some countries due to difficulties experienced with vaccine supply, virus mutations, and the relaxation of the restrictions due to a decrease in the number of infections. The pandemic has physical, psychological, and social effects on individuals. While physical problems are at the forefront in the initial stages of spread, psychological and social problems continue to significantly impact individuals in the later stages of the pandemic. These problems can occur even in individuals who are not at high risk of getting sick [22, 28].

Traumatic events can reduce people’s sense of security, increase levels of existential dread, and adversely affect their psychological well-being. Uncertainty surrounding the duration of the pandemic, constant streams of pandemic information, reduced social contact, and government-imposed lockdowns negatively affect the mental health of individuals. Symptoms such as anxiety, depression, fear, stress and sleep deprivation have been common during the COVID-19 pandemic [32].

Although not pervasive as COVID-19, mental health problems emerged in healthcare professionals, the general population, and victims of severe acute respiratory syndrome (SARS) or middle east respiratory syndrome (MERS) during the SARS and MERS epidemics [7, 11, 17]. Post-traumatic stress disorder (PTSD) and depressive disorders have been identified as the most common long-term mental health problems in individuals affected by SARS. Similar results were reported in a study related to the MERS outbreak [17]. These results suggest that the COVID-19 pandemic could have psychological and social impacts on patients infected with COVID-19, the general population, and healthcare workers [10].

The psychological consequences of the COVID-19 pandemic are already evident in the stresses associated with risk of infection, quarantine, self-isolation and traumatic experiences in families and communities. The pandemic and subsequent social distancing measures may beget feelings of loneliness, hopelessness, and existential dread—independent predictors of suicide. The COVID-19 pandemic can be stressful for individuals and communities. Fear and anxiety about an illness can be overwhelming and generate strong emotions in adults and children [18, 33]. Individuals tend to feel anxious and insecure when the environment changes. When an infectious disease’s cause, progression, and consequences are unclear, rumours propagate, and closed-minded attitudes emerge. Fear has been a known and common response to contagious epidemics for centuries, e.g. the plague. People respond to such threats in many individualised ways. Fear of the unknown increases anxiety in healthy individuals and those with pre-existing mental health problems. The spread of disease and its impact on people, health, hospitals and economies is one such unknown. Pandemics cause individuals, families, and communities to experience feelings of hopelessness, despair, grief and a profound loss of meaning [18].

Isolation strategies to prevent the spread of the virus have caused psychological and social problems by closing schools and workplaces, decreasing autonomy, and causing financial and safety concerns [28, 36]. These strategies have led to loneliness, anxiety, and depressive symptoms by restricting access to social support systems such as family or friends [28]. Social isolation, quarantine, social and economic changes caused by the pandemic have triggered emotions that mediate psychological problems such as sadness, anxiety, fear, stress, disappointment, guilt, helplessness, loneliness and anger. These feelings are typical features of mental health problems experienced during or after a crisis [1, 3, 19]. Consistent exposure to pandemic-related information on social media during this crisis has also led to mental health problems [19, 38].

Patients infected by COVID-19 are the most affected group. These individuals experience additional stressors such as fear of infecting family, social stigma, and coping with difficult treatment processes alone [28]. These stressors can have long-term effects on individuals diagnosed with COVID-19 who require treatment in addition to the financial burden of managing the disease [24].

The rise in the number of people hospitalised with COVID-19 has increased the workloads of healthcare workers, worsening working conditions. Lai et al. [16] state that the exponential increase in the number of cases, workload, personal protective equipment (PPE) limitations, sensationalist media, lack of medication, and insufficient support, can have a physical and psychological impact on healthcare workers [16].

Recent publications on COVID-19 showed that researchers focus on epidemiology, clinical features, radiology findings, and treatment; very few studies have focused on the mental health of those affected by the disease [13, 28]. Studies on the psychological effects of the pandemic were restricted to healthcare workers [8, 16] and the general population [34].

The most important psychological effects of the pandemic are anxiety and depressive symptoms in the short term. It follows that the general population, infected individuals, and healthcare workers on the frontline of the pandemic experience similar psychosocial problems.

Increased psychological distress has been reported predominantly in the general population, frontline healthcare workers, and individuals recovering from COVID-19. There are few studies on the mental health of COVID-19 inpatients, while many studies have been conducted on the mental health of frontline healthcare workers and general populations affected by the pandemic [12, 26]. There may be a difference in anxiety and depressive symptoms between these populations. Moreover, there is limited research on the psychological distress (anxiety, depression, etc.) of patients with COVID-19.

Therefore, this study aimed to determine the level of anxiety and depressive symptoms of patients hospitalised for COVID-19, frontline healthcare workers, and the general population during the first wave of the COVID19 pandemic in Istanbul. This study also examined the effect of factors potentially affecting these variables, such as age, gender, marital status, physical or psychiatric illness, etc.

## Methods

### Study design and sample

The present study used a descriptive, cross-sectional survey design. The data were collected from June 31 to July 15, 2020, during the first pandemic wave in Istanbul, Turkey.

The sample of the study consisted of the general population (*n* = 162), frontline healthcare workers (*n* = 131) and COVID-19 inpatients (*n* = 86). Data were collected through online surveys from frontline healthcare workers and the general population via social media using convenience sampling. The target population for the electronic survey were frontline health care workers and the general population over 18 years old, living in İstanbul. Individuals agreeing to participate were asked to complete the questionnaire through social media (WhatsApp, Twitter and Facebook).

The convenience sampling method was used to obtain data from patients with COVID-19 treated in a training and research hospital in Istanbul. Data were collected from patients hospitalised with COVID-19 who agreed to participate in the study and were able to fill out the health-status data-collection form. Informed consent was obtained before data collection.

### Data collection tools

The data were collected using the Individual Characteristics Form, GAD-7 and BDI.

*Individual Characteristics Form* consists of common questions about participants’ age, gender, marital status, employment status, whether they have chronic physical or mental illness, and whether they have experienced a significant life event in the past year. Questions unique to the different sample groups were prepared.

For the general population, participants were asked about: whether they were in quarantine, whether their relatives had been diagnosed with COVID-19, their levels of fear or anxiety of being infected with COVID-19, their fear of transmitting it to people they are close to.

COVID-19 inpatient participants were asked about: how many days they had been in hospital, fear of re-hospitalisation with COVID-19, their fear of infecting people they are in contact with.

Frontline healthcare worker participants were asked about: whether their PPE was sufficient, whether there was a change in accommodation, whether they or their relatives had been diagnosed with COVID-19, their fear or anxiety levels of being infected with COVID-19, their fear of infecting the people they are in contact with.

*Generalised Anxiety Disorder Scale (GAD-7)* was developed by Spitzer et al. [30] and translated to Turkish by Konkan et al. [14]. It consists of 4-Point Likert scale type questions (0—not at all, to 3—almost every day) for seven items. It was evaluated generalised anxiety symptoms. The scores of 5, 10 and 15 obtained in the scale are cut-off points for mild, moderate and severe anxiety, respectively. GAD7 is a valid and efficient scale. Cronbach’s alpha of the current study was 0.89.

*Beck Depression Inventory (BDI)* was developed by Beck (1961) for evaluating depression symptoms in four areas: emotional, cognitive, vegetative and motivational. It was translated to Turkish by Hisli (1989). This scale consists of 4-point Likert scale-type questions for 21 items. The scores obtainable are between 0–63. The cut-off point for the Turkish sample was 17. For BDI: 0–9 points, minimal depressive symptoms; 10–16 points, mild depressive symptoms; 17–24 points, moderate depressive symptoms; 25 and above points, severe depressive symptoms. The BDI is a valid and efficient scale. Cronbach’s alpha of the current study was 0.89.

### Statistical analysis

Data analyses were run via Statistical Package for the Social Sciences version, 20.0. Average and standard deviation for continuous variables were calculated. Frequency and percentage values for categorical variables were calculated. The normality distribution of the data was evaluated using the Kolmogorov–Smirnov and skewness–kurtosis values. Skewness and kurtosis values in the data with a sample larger than 30 ± 2 indicate that the data are normally distributed [31]. The total BDI and GAD-7 scores were obtained for the mean difference statistics. The one-way ANOVA test (*F* table value) was used to compare the means of three or more independent groups. Levene’s test statistic was evaluated for homogeneity of variance between the groups. The Bonferroni correction was used for different statistically significant variables for a dual comparison of three or more groups according to the homogeneity of variance. The Chi-square (*χ*^2^) test was used to compare categorical variables. Pearson correlation and multiple regression analysis were used to analyse the relationships between the averages.

## Results

The study participants were divided into COVID-19 inpatients, frontline healthcare workers, and the general population. Three hundred seventy-nine participants—162 (42.7%) general population, 131 (34.6%) frontline healthcare workers, and 86 (22.7%) COVID-19 inpatients—completed surveys. The individual characteristics of the participants are shown in Table 1.

**Table 1:** The individual characteristics of participants

Variables	General population	Frontline health workers	Covid-19 inpatients	*F*/χ^2^	*p*
Total *N* by group	162	131	86		
^a^Mean age M ± *SD*	36.28 ± 11.20 (18–66)	29.75 ± 7.35 (20–56)	38.27 ± 15.87 (20–78)	18.122	< 0.001
^b^Gender *n* (%)					
Female	119 (73.5)	114 (87)	24 (27.9)	87.253	< 0.001
Male	43 (26.5)	17(13)	62 (72.1)		
^b^Marital status *n* (%)					
Married	85 (52.5)	45 (34.4)	43 (50)	10.433	< 0.01
Single	77 (47.5)	86 (65.6)	43 (50)		
^b^Working status *n* (%)					
Working	109 (67.3)	131 (100)	49 (57)	390.93	< 0.001
Retired	10 (6.2)	–	7 (8.1)		
I quit my job due to the epidemic	43 (26.6)	–	30 (34.9)		
^b^Having a physical *n* (%)					
Yes	22 (13.6)	15 (11.5)	24 (27.9)	11.736	< 0.01
No	140 (86.4)	116 (88.5)	62 (72.1)		
^b^Important life event *n* (%)					
Yes	43 (26.5)	43 (32.8)	5 (5.8)	21.754	< 0.001
No	119 (73.5)	88 (67.2)	81 (94.2)		
*Being sufficient of personal protective equipment *n* (%)					
Yes		96 73.3			
No		35 26.7			
Being infected with Covid-19 *n* (%)					
Yes	2 (1.2)	9 (6.9)			
No	160 (98.8)	122 (93.1)			
*Being in quarantined *n* (%)					
Yes	93 (57.4)				
No	32 (19.8)				
Only banned days	37 (22.8)				
*Profession *n* (%)					
Nurse		121 (92.4)			
Physician		5 (3.8)			
Other		5 (3.8)			
*Accommodation areas during the outbreak *n* (%)					
Own home		92 (70.2)			
Hotel/lodging/other home		32 (24.4)			
Isolation in my own home		7 (5.3)			
Emotions *M* ± *SD*					
^a^Do you have anxiety/fear of infecting your relatives or people you contact with?	6.67 ± 2.68	8.35 ± 2.30	6.77 ± 3.66	14.601	< 0.001
^a^Do you have anxiety/fear of being infected?	6.114 ± 2.827	5.6605 ± 2.500		− 1.457	0.146
*Do you have anxiety/fear of re-hospitalisation with diagnosis of Covid-19?			3.16 ± 3.18		

^*^Questions were asked only to one group. *F*: one-way ANOVA test; *χ*^2^: Chi-square test

^a^One-way anova was used for compare the means of three or more independent groups

^b^The Chi-square (*χ*^2^) test was used for comparison of categorical variables

Frontline healthcare workers were the youngest, COVID-19 inpatients, the oldest group. Frontline healthcare workers’ fear of infecting their relatives was significantly higher than the general population (*p* < 0.001). There was no significant difference between health workers and the general population regarding their fear of infection.

### Individual participant characteristics

Table 2 indicates participants’ mean Beck Depression Score and General Anxiety Disorders Scale. Frontline healthcare workers had the highest mean score on the Beck Depression Scale (15.64 ± 9.95) and General Anxiety Disorder Scale (8.52 ± 5.01). Increased prevalence of depression (70.2%) and anxiety (76.3) was also found, predominantly in healthcare workers (*p* < 0.001). Moreover, COVID-19 inpatients and the frontline healthcare workers were more likely to exhibit anxiety (*p* < 0.001) compared to the general population (Table 2).

**Table 2:** The GAD-7 and BDI findings of participants

Variables	General population	Frontline health workers	Covid-19 inpatients	*F*/*χ*^2^	*p*
Prevalence of anxiety	103 (63.6%)	100 (76.3%)	30 (34.9%)	38.196	< 0.001
Prevalence of depression	75 (46.3%)	92 (70.2%)	29 (33.7%)	31.039	< 0.001
GAD7 *M* ± SD	6.70 ± 4.89 (0–20)	8.52 ± 5.01 (0–21)	4.5 ± 4.66 (0–20)	17.711	< 0.001
Beck *M* ± SD	10.95 ± 8.77 (0–36)	15.64 ± 9.95 (0–42)	8.54 ± 7.50 (0–40)	18.374	< 0.001
Severity of anxiety symptoms *n* (%)					
Normal	59 (36.4)	31 (23.7)	56 (65.1)	42.110	< 0.001
Mild	63 (38.9)	49 (37.4)	18 (20.9)		
Middle	27 (16.7)	33 (25.2)	7 (8.1)		
Severe	13 (8)	18 (13.7)	5 (5.8)		
Severity of depression symptoms *n* (%)					
Normal	87 (53.7)	39 (29.8)	57 (66.3)	41.359	< 0.001
Mild	49 (30.2)	44 (33.6)	20 (23.3)		
Middle	16 (9.9)	38 (29)	7 (8.1)		
Severe	10 (6.2)	10 (7.6)	2 (2.3)		

*F* = one-way ANOVA, *χ*^2^ = Chi-square test

### GAD-7 and BDI findings of participants

The independent *t*-test samples were conducted to examine whether the depression and anxiety levels of the participants differ according to the individual characteristics shown in Table 3. It was observed that there was no significant difference between the age and physical illness of participants and their depression and anxiety levels.

**Table 3:** The comparison and relationship of BDI, GAD-7 with individual characteristics of participants

	GAD-7			BDI		
	General population	Frontline health worker	Covid-19 inpatients	General population	Frontline health worker	Covid-19 inpatients
Gender						
Female	7.34 ± 4.83	8.44 ± 5.14	5.91 ± 5.85	11.35 ± 8.62	15.32 ± 10.36	11.45 ± 9.69
Male	4.95 ± 4.68	9.05 ± 4.17	3.95 ± 4.04	9.86 ± 9.17	17.76 ± 6.42	7.41 ± 6.20
*t*/*p*	2.803; < 0.01	− 0.467; 0.641	1.772; 0.141	0.956; 0.341	− 1.329; 0.194	1.896; 0.067
Marital status						
Married	6.12 ± 5.00	8.17 ± 5.45	4.79 ± 4.62	9.83 ± 8.78	13.08 ± 10.59	8.48 ± 7.24
Single	7.35 ± 4.72	8.70 ± 4.79	4.20 ± 4.75	12.19 ± 8.65	16.97 ± 9.39	8.60 ± 7.85
*t*/*p*	− 1.593; 0.113	− 0.574; 0.567	0.575; 0.567	− 1.720; 0.087	− 2.152; < 0.01	− 0.071; 0.943
Having physical illness						
Yes	7.90 ± 4.58	9.33 ± 5.16	5.45 ± 5.60	11.81 ± 9.11	17.4 ± 10.6	11.41 ± 9.61
No	6.52 ± 4.93	8.42 ± 5.01	4.12 ± 4.24	10.82 ± 8.74	15.41 ± 9.89	7.43 ± 0.26
*t*/*p*	1.238; 0.217	0.660; 0.510	1.187; 0.239	0.494; 0.621	0.726; 0.469	2.258; 0.070
Important life event						
Yes	8.58 ± 5.66	9.37 ± 5.08	4.20 ± 3.03	13.95 ± 10.35	17.44 ± 9.82	7.40 ± 4.15
No	6.03 ± 4,.42	8.11 ± 4.96	4.51 ± 4.76	9.87 ± 7.89	14.76 ± 9.95	8.61 ± 7.67
*t*/*p*	2.671; < 0.01	1.352; 0.179	− 0.147; 0.883	2.663; < 0.01	1.453; 0.149	− 0.350; 0.727
Age	r: − 0.082	r: − 0.161	r: 0.103	r: − 0.136	r: − 0.093	r: 0.069
Fear of infecting relatives	r: 0.406**	r: 0.351**	r: 0.123	r: 0.356**	r: 0.288**	r: 0.243*
Fear of being infected	r: 0.422**	r: 0.319**		r: 0.322**	r: 0.188*	
Days of hospitalisation			r: − 0.263*			r: − 0.138
Fear of re-hospitalisation			r: 0.356**			r: 0.342**

*t*: independent groups *t* test, *r*: Pearson rho correlation coefficient, *p < 0.05; **p < 0.01

In the general population, the anxiety levels of female participants were higher compared to male participants (*t: 2.803; p* < *0.01*). There was no significant association between anxiety and depression levels and gender in the other groups. Moreover, the depression levels of single health workers were higher compared to married workers (*t*: *− 2.152; p* < *0.01*).

In the general population, depression and anxiety levels of participants who had a significant life event were significantly higher than those who did not have one. The anxiety (*t: 2.671; p*<*: 0.01*) and depression (*t: 2.663*; *p* < *0.01*) levels of participants in the general population who experienced a significant life event in the past year were found to be significantly higher than those who did not have one. Covid-19 inpatients in the 1st days of hospitalisation, with a high possibility of re-hospitalisation, have higher anxiety levels.

While the anxiety levels of frontline healthcare workers and the general population highly correlated with the fear of infecting others, no statistically significant relationship was found between COVID-19 inpatient anxiety and their fear of infecting relatives (*p* < *0.001*).

Moreover, there was a significant positive relationship between depression levels of participants and fear of infecting their relatives in all groups (*p* < *0.001*). Furthermore, COVID-19 inpatients with high fear of re-hospitalisation have higher BDI scores.

### The comparison and correlation of BDI and GAD-7 with individual characteristics of participants

Multiple linear regression analysis was performed to determine the individual characteristics affecting the participants’ depression and anxiety levels (Table 4). Multiple regression analysis results were significant (*p* < *0.001*). When the beta values in the table are examined, and all independent variables are included in the regression model, gender (*β* = *0.170, p* = *0.019*), significant life events (*β* = *151, p* = *0.038*), and fear of infecting relatives (*β* = *0.355, p* = *0.000*) contribution significantly to the reasons for anxiety in the general population. This result explains the 20% variance in anxiety level. In the general population, it was determined that fear of infecting relatives contributed significantly to the level of depression (*p* < *0.001*). This result explains the 12% variance in depression levels.

**Table 4:** Results of multiple linear regression analysis^a^ on factors significantly associated with depression and anxiety

	Independent variables	GAD7						BDI					
		*B*	95% CI	*β*	*t*	Sig	*f*	*B*	95% CI	*β*	*t*	Sig	*f*
General population	Age	0.001	(− 0.067/0.069)	0.002	0.028	0.978	7.810	− 0.044	− 0.171/0.083	− 0.056	− 0.684	0.495	4.958
	Gender (female vs male)	1.874	0.314/3.434	0.170	2.373	0.019		0.649	− 2.273/3.572	0.033	0.439	0.661	
	Marital status (married vs single)	− 0.738	− 2.229/0.753	− 0.076	− 0.978	0.330		− 1.404	− 4.197/1.388	− 0.080	− 0.994	0.322	
	Living important life event (yes vs no)	1.668	0.092/3.244	0.151	2.091	0.038		2.729	− 0.223/5.682	0.138	1.826	0.070	
	Having physical Illness (yes vs no)	− 1.030	− 3.093/1.034	− 0.072	− 0.985	0.326		− 1.061	− 4.927/2.806	− 0.042	− 0.542	0.589	
	Fear of infecting relatives	0.647	0.383/0.912	0.355	4.831	0.000		1.009	0.514/1.505	0.309	4.021	0.000	
	*R*^2^_adj_ = 0.20 (*N* = 165, *p* = 0.000). CI = confidence interval for *B*							*R*^2^_adj_ = 0.12 (*N* = 165, *p* = 0.000) CI = confidence interval for *B*					
Frontline healthcare workers	Age	− 0.039	− 0.166/0.088	− 0.057	− 0.612	0.542	5.054	0.129	− 0.126/0.383	0.095	1.001	0.319	4.616
	Gender (female vs male)	0.013	− 2.391/2.417	0.001	0.011	0.992		− 1.247	− 6.065/3.570	− 0.042	− 0.512	0.609	
	Marital status (married vs single)	− 0.164	− 2.068/1.740	− 0.016	− 0.170	0.865		− 4.569	− 8.385/− 0.753	− 0.219	− 2.370	0.019	
	Living important life event (yes vs no)	1.502	− 0.204/3.207	0.141	1.742	0.084		2.589	− 0.830/6.007	0.123	1.499	0.136	
	Having physical Illness (yes vs no)	− 0.423	− 2.996/2.150	− 0.027	− 0.325	0.745		− 1.537	− 6.693/3.619	− 0.049	− 0.590	0.556	
	Fear of infecting relatives	0.687	0.326/1.048	0.315	3.767	0.000		1.286	0.563/2.009	0.298	3.519	0.001	
	Lack of protective equipment’s (yes vs no)	3.045	1.224/4.865	0.270	3.310	0.001		5.398	1.750/9.047	0.241	2.929	0.004	
	*R*^2^_adj_ = 0.179 (*N* = 130, *p* = 0.000). CI = confidence interval for *B*							*R*^2^_adj_ = 0.163 (*N* = 130, *p* = 0.000). CI = confidence interval for *B*					
Covid-19 inpatients	Age	− 0.011	− 0.106/0.084	− 0.036	− 0.223	0.824	2.410	− 0.046	− 0.197/0.105	− 0.098	− 0.610	0.543	2.553
	Gender (female vs male)	0.782	− 1.686/3.251	0.076	0.631	0.530		2.373	− 1.549/6.295	0.143	1.205	0.232	
	Marital status (married vs single)	0.184	− 2.315/2.684	0.020	0.147	0.884		− 0.268	− 4.240/3.705	− 0.018	− 0.134	0.894	
	Living important life event (yes vs no)	0.674	− 3.426/4.775	0.034	0.327	0.744		0.645	− 5.881/7.172	0.020	0.197	0.844	
	Having physical Illness (yes vs no)	− 0.676	− 3.311/1.959	− 0.065	− 0.511	0.611		− 3.292	− 7.488/0.903	− 0.198	− 1.563	0.122	
	Fear of infecting relatives	0.077	− 0.197/0.352	0.061	0.561	0.576		0.344	− 0.093/0.782	0.168	1.567	0.121	
	Days of hospitalisation	− 0.222	− 0.473/0.030	− 0.191	− 1.752	0.084		− 0.143	− 0.545/0.258	− 0.077	− 0.712	0.479	
	Fear of re-hospitalisation	0.444	0.132/0.757	0.303	2.832	0.006		0.576	0.060/1.093	0.244	2.221	0.029	
	*R*^2^_adj_ = 0.10 (*N* = 85, *p* = 0.027). CI = confidence interval for *B*							*R*^2^_adj_ = 0.12 (*N* = 85, *p* = 0.16) CI = confidence interval for *B*					

*GAD7* Generalised Anxiety Disorder Scale, *BDI* Beck Depression Inventory

^a^Enter model was applied

According to the multiple regression analyses, lack of PPE and the fear of infecting relatives explained 17% of the anxiety variance level and 16% of the depression variance level among frontline health care workers.

COVID-19 inpatients’ fear of being hospitalised again was a significant predictor of anxiety and depression (*p* < *0.005*). This result explained 10% of the variance in the anxiety level and 12% of the variance in the depression level.

## Discussion

The levels of depression, anxiety, and related factors in the three groups (general population, frontline healthcare workers, COVID-19 inpatients) during the first wave of the pandemic in Istanbul, Turkey, was investigated in this study. Studies on this subject have increased but were limited, particularly in Turkey. This study showed that the COVID-19 pandemic has negatively affected individuals’ mental health, with frontline healthcare workers needing particular attention due to psychological distress.

The prevalence of anxiety in the general population, frontline healthcare workers, and COVID-19 inpatients were 63.6%, 76.3%, 34.9%, respectively. The prevalence of depression was approximately 46.3%, 70.2%, 33.7%, respectively. These results were higher than those found in other countries [2, 10, 23]. In China, it was shown that 8.3% of participants had anxiety in the study conducted with affected and unaffected people [16]. The depression prevalence was also lower than our results in this study. It was also found that severe and extremely severe levels of anxiety and depression in the Spain sample were lower than in this study [23]. Another study was conducted in Malaysia to determine depression and anxiety levels during the 3rd wave of the pandemic. The prevalence of depression was 87.7%, and the prevalence of anxiety was 43.6% [20]. In a review of 13 studies examining the symptoms of anxiety and depression in healthcare workers during the pandemic, anxiety was assessed with a pooled prevalence of 23.2%. Depression was assessed in 10 studies, with a prevalence rate of 22.8% [25]. It is noteworthy that the study was carried out just before the normalisation phase of the outbreak in Turkey. Possible reasons for these differences are as follows. Firstly, the study was carried out in Istanbul, the city with the highest number of cases and a prolonged outbreak. COVID-19 had spread globally and restriction measures implemented by governments may have affected these results. Secondly, knowledge of infectious diseases is a factor. The level of knowledge affects reactions to a crisis, particularly in a pandemic [15]. Turkish people did not know how to cope with a crisis of this scale. Use of different measurement instruments, different phases of the pandemic, different study designs, and cultural backgrounds could be a reason for these variable results.

Our study shows high levels of anxiety and depression during the COVID-19 outbreak, particularly in frontline healthcare workers and the Turkish public. When we compare the average values between the three groups, healthcare workers have greater levels of anxiety and depression than other groups. This is contrary to the results of the large sample study in China [10]. Our results suggest that anxiety and depression levels of frontline healthcare workers increase when a major infectious disease pandemic occurs. In a study conducted in Turkey before the COVID-19 pandemic, the frequency of depression was 29% among doctors employed in emergency units [5]. In another study conducted during the COVID-19 pandemic, 13.7% of the participants showed symptoms of depression, and 26.7% of those exhibited symptoms of generalised anxiety [37].

In contrast, another study comparing the depression and stress levels of healthcare and non-healthcare workers in Turkey found no difference in the stress and depression levels of the participants [4]. It is thought that this difference in results was because our study was conducted in the first wave of the pandemic. It is believed that the availability of the vaccine, the decrease in the number of COVID-19 inpatients, and the experience of healthcare professionals managing the pandemic have progressed since then.

Similar to the psychological consequences of previous epidemics such as SARS [29], we found that approximately 3/4 of the frontline healthcare workers exhibited symptoms of anxiety and depression. This study showed no difference between frontline healthcare males’ and females’ depression and anxiety levels. This differs from previous research indicating that women were more likely to suffer from depression and anxiety than men [6, 9]. However, this study also found that the anxiety levels of female participants in the general population were higher than men.

Contrary to previous research conducted in other countries, there was no relationship between age, anxiety, and depression levels [10, 23, 29]. To slow the spread of COVID-19, the Turkish government imposed stringent restrictions on individuals under 20 and over 65 years of age which could affect this result.

There were high levels of depression among single (uninvolved romantically) frontline healthcare workers in our study. Similarly, Marzo et al. [20] found that being young, single, and female was a predictor of depression and anxiety [20]. This may be related to the lack of social support systems, living away from home during the pandemic, and not communicating due to fear of transmitting the disease to their relatives.

There was a positive correlation between fear of infecting relatives and anxiety and depression in the general population and frontline healthcare worker groups. There was also a positive correlation between fear of becoming infected and levels of anxiety and depression in these subgroups. Working with suspected positive patients, contact with confirmed infection cases, and a lack of PPE, increased the risk of contracting COVID-19 for frontline healthcare workers. Additionally, healthcare workers worried more about infecting family members, relatives and friends due to working with infected patients. These emotional challenges caused anxiety and depression among the healthcare workers [10, 27]. The physical health implications of contracting COVID-19 or transmitting it to someone else lead to anxiety and depression in the general population.

There was a high level of anxiety during the 1st days of hospitalisation in patients infected with COVID-19. There was also a relationship between the fear of re-infection and levels of anxiety and depression. Possible reasons for these mental health problems facing infected patients could be confronting an unknown disease, treatment in isolation, and physical complications of COVID-19 [28, 38].

There were some limitations to this study. The first was that cross-sectional designs and self-reported data do not allow for confident causal conclusions. The second was that the method of purposive sampling and online surveys could lead to bias, which cannot be measured or controlled for. Therefore, results from the data cannot be generalised throughout Turkey. The strength of this study was in comparisons between the mental health outcomes among three subgroups during COVID-19 surges and factors related to levels of anxiety and depression.

## Conclusions

It is important to diagnose and treat psychiatric conditions that occur in individuals in the future. Consideration must be given to the pandemic’s negative impact on mental health to reduce the mental burden of the disease. Future research should investigate a larger sample across different ages, genders, and job roles, particularly other frontline workers like teachers, pharmacists, retail workers, etc. Intervention studies that seek to improve the mental health of individuals are also recommended. Multidisciplinary teams—consisting of psychiatrists, psychiatric nurses, clinical psychologists, and other mental health professionals—should be formed by government and health authorities to meet the psychological support needs of individuals.

## Data Availability

The data sets used and/or analysed during the current study are available from the corresponding author on reasonable request.

## Abbreviations

WHO: World Health Organization
SARS: Severe acute respiratory syndrome
MERS: Middle east respiratory syndrome
PTSD: Post-traumatic stress disorder
GAD-7: Generalised Anxiety Disorder Scale
BDI: Beck Depression Inventory
PPE: Personal protective equipment
